# Gendered Barriers and Opportunities for Women Smallholder Farmers in the Contagious Caprine Pleuropneumonia Vaccine Value Chain in Kenya

**DOI:** 10.3390/ani12081026

**Published:** 2022-04-14

**Authors:** Kitoga Byalungwa Kyotos, Jemimah Oduma, Raphael Githaiga Wahome, Catherine Kaluwa, Faduma Abdulahi Abdirahman, Angela Opondoh, Jeanette Nkatha Mbobua, John Muchibi, Brigitte Bagnol, Meghan Stanley, Marieke Rosenbaum, Janetrix Hellen Amuguni

**Affiliations:** 1 Department of Animal Production, University of Nairobi, Nairobi 00100, Kenya; luckykitoga@gmail.com (K.B.K.); rgwahome@uonbi.ac.ke (R.G.W.); fadumo.abdullaahi@gmail.com (F.A.A.); 2 Department of Veterinary Anatomy and Physiology, University of Nairobi, Nairobi 00100, Kenya; joduma@uonbi.ac.ke (J.O.); ckaluwa@uonbi.ac.ke (C.K.); 3 Institute of Anthropology, Gender and African Studies, University of Nairobi, Nairobi 00100, Kenya; angellaopondoh@gmail.com (A.O.); jynettenkatha@gmail.com (J.N.M.); 4 Fairdeal Agrivet and Services, Nairobi 00100, Kenya; jmuchibi@gmail.com; 5 Department of Anthropology, University of the Witwatersrand, Johannesburg 2000, South Africa; bagnolbrigitte@gmail.com; 6 International Rural Poultry Centre (IRPC), Kyeema Foundation, Brisbane City, QLD 4000, Australia; 7 Cummings School of Veterinary Medicine, Tufts University, North Grafton, MA 01536, USA; meghan.stanley@tufts.edu (M.S.); marieke.rosenbaum@tufts.edu (M.R.)

**Keywords:** women smallholder farmers, gender, small ruminants, contagious caprine pleuropneumonia, livestock vaccine value chain

## Abstract

**Simple Summary:**

Small animals such as goats, sheep and chickens are an important source of income for rural livelihoods, especially for women farmers in Africa, because they are able to control the resources that come from the sale of these animals. However, one of the biggest problems they face is livestock diseases, even when vaccines are available. In Kenya, Contagious Caprine Pleuropneumonia (CCPP) is a highly infectious disease of goats with a mortality rate of more than 70%. A vaccine for CCPP is available but difficult to access by women in the rural areas. This study examines the gaps and barriers that prevent women smallholder farmers from effectively accessing and adopting CCPP vaccination for their animals in the Machakos district of Kenya. Our results indicate that key constraints to vaccine access and adoption for rural smallholder women farmers are lack of a cold chain for vaccine maintenance, inadequate and late delivery of veterinary services, lack of information and training, and limited financial capacity to purchase the vaccine. If more resources, information, and training is made available to women smallholder farmers through government or the private sector, there would be improved livestock productivity, better livelihoods, and increased opportunities and agency for women.

**Abstract:**

Most rural women smallholder farmers in Kenya generate income from the sale of small ruminant animals. However, diseases such as Contagious Caprine Pleuropneumonia (CCPP) prevent them from optimizing earnings. A crucial aspect for the control of CCPP is vaccination. In Kenya, CCPP vaccines are distributed through a government delivery mechanism. This study examines gaps and barriers that prevent women smallholder farmers from accessing CCPP vaccines. Qualitative data collection tools used were focus groups discussions, focus meals, jar voices and key informant interviews. Using outcome mapping (OM) methodology, critical partners and stakeholders in the CCPP vaccine value chain (CCPP-VVC) were identified to be the manufacturers, importers, distributors, agrovets, public and private veterinarians, local leaders, and farmers. Respondents highlighted the barriers to be limited access to vaccines due to cold chain problems, inadequate and late delivery of services, lack of information and training on vaccines, and financial constraints. Identified opportunities that can support women’s engagement in the CCPP-VVC are the Kenya Governments two-third gender rule, which requires that not more than two thirds of the members of elective or appointive bodies shall be of the same gender, and positive community perception of female veterinarians. We conclude that more resources and training should be made available to women farmers, and that gender perspectives on policy development related to livestock production and disease prevention are urgently needed to improve livestock productivity and increase agency for women.

## 1. Introduction

According to the FAO (2018), the livestock sector is an important source of livelihood for about 1.7 billion people worldwide [1]. In marginal rural areas, where poverty is rampant, livestock represent an important asset for local, cultural, and socio-economical systems, and allow the effective use of otherwise non-utilizable resources [2]. In Africa, small ruminants, such as goats, are considered one of the assets that women can possess, take control over, and sell to meet their financial needs. Goats have a comparative advantage of short gestation periods and high incidences of multiple births [3]. They play a critical role in rural households, providing nutritious food sources such as milk and meat, are a source of income and savings, and are used in traditional and cultural functions [4]. In many cases, goats are a significant component of smallholder risk management strategies [5]. Small livestock products meet the needs of rural women, as they require less inputs/investments and can be managed even with limited access to land.

Women play a central role in most countries as food producers and providers [6], and control (some) livestock products that are essential for food and nutrition security [7]. Women constitute 70% of food producers and providers in Kenya and represent the majority of livestock keepers [8]. Raising livestock, as opposed to crops, tends to be a more accessible agricultural pursuit for women and, as a result, they rely on their animals more heavily than their male counterparts [9]. Studies have shown that female livestock keepers tend to own more small ruminants (goats, sheep, among others) and poultry than large livestock (water buffalo and cows) [10]. Such studies find that women’s contributions, while crucial, are hidden and given low social recognition, while men are predominant actors in the most lucrative activities and nodes, where profits and social connections usually abound [7].

The productivity of small ruminants is constrained by preventable livestock diseases. Contagious caprine pleuropneumonia (CCPP) is one of the most prevalent infectious diseases affecting small ruminants, with unfavorable outcomes and serious consequences on people’s livelihoods and economies [11]. Globally, the disease has been reported in 38 African and Asian countries [12], where it is endemic and is a major threat to the goat farming industry [13]. This is so despite the availability of a vaccine. CCPP is a highly infectious disease caused by *Mycoplasma capricolum* sub spp. *capripneumoniae* (Mccp) [14,15]. It is listed as a notifiable disease by the World Organization of Animal Health (OIE) with implications for international trade [16]. Transmitted by direct and close contact between animals, it has a greater than 70% mortality rate and a morbidity rate between 80% and 100% [17].

In Kenya, the government implements bi-annual vaccination campaigns against CCPP, but only in pastoral areas where the disease is endemic. In other parts of Kenya such as Machakos County, government-led vaccination is only done in case of a reported outbreak. However, individual farmers can also access the vaccine through private veterinary service providers outside the government-led vaccination programs, but at a cost. The vaccine used, CaprivaxTM, is an inactivated vaccine produced locally by the Kenya Veterinary Vaccines Production Institute (KEVEVAPI). Even though the vaccine efficacy is estimated at 95% [18], other factors interfere with the vaccination effectiveness. The vaccine needs to be stored at 2–8 °C, requiring a well-maintained cold chain. CCPP outbreaks can be devastating to families with limited resources. One goat can infect a full herd in days, leading to the loss of entire herds, and risking the livelihoods of the whole family.

Women’s access to vaccines, and information and training in modern livestock disease management is indirect, and mostly through men, lowering their involvement and efficiency [19]. Women also lack access to livestock services and product delivery systems, which are male dominated [7]. Empowering female farmers, especially rural subsistence farmers, has been shown to be an effective means of fighting household hunger and poverty. Interventions that ensure women’s access to CCPP and other livestock vaccines by reducing gender-related barriers to their active participation along the VVC as service providers, distributors, users, and overall beneficiaries, have the potential to empower the women and lead to improved livestock productivity. Such interventions can make women more visible and foster open discussions about gender roles at the household and community level, potentially improving their economic and social status, as well as their positions in production systems [20].

Any mediation, therefore, that aims to improve livestock health of small ruminants through vaccination is expected to offer direct and great benefits to women small-scale farmers, and application of the vaccines by women farmers could improve animal health with better reproductive and productive potential. This empowers women and increases their economic potential and agency [21,22].

Studies have shown that the vaccines do not reach smallholder farmers [23,24], and this is generally blamed on the low level of involvement of farmers in vaccination [25]. The situation is a result of many factors, including lack of awareness of farmers about the benefits of vaccination, poor strategies of vaccination campaigns and low interaction among the vaccine chain stakeholders [26]. A well-designed vaccine value chain has a positive influence on vaccination efforts by dropping costs and enhancing coverage [27]. Global health actors and agencies have thus expressed the need for all vaccine supply chains, including livestock vaccines, which are often ignored, to function at their best levels [28].

Many development interventions now utilize the value chain approach as an important entry point for engaging smallholder farmers, individually or collectively [29]. A value chain is described as a portrayal of a firm’s value-adding actions, based on its pricing strategy and cost structure, and emphasizing the interconnections and associations between and within actors as well as the governance and relationships between actors in the creation of value for a firm [30]. There are four main components in a traditional value chain analysis [31]: (i) a mapping and characterization of the actors involved in the chain from production, distribution, and delivery of a particular product to the end user; (ii) an evaluation of governance and coordination systems and practices that exist between actors, to recognize the institutional arrangements that may need to be targeted to improve capacities, correct distributional biases, and build up value; (iii) an analysis of opportunities for progression within the chain by different actors, and (iv) assessment of benefit sharing among actors in the chain to determine who benefits from participation in the chain and which actors could benefit from increased support or organization.

An analysis of the CCPP vaccine value chain (CCPP-VVC) from a gendered perspective can assist in identifying bottlenecks in the entire system, and specifically places where women’s participation is low, allowing strategic interventions for women’s inclusion and promotion of gender equality. The value chain runs from vaccine manufacturing, through distribution and delivery, all the way to the livestock farmer/end user including the policy and regulatory context [32]. A gendered Livestock VVC (LVVC) analysis helps to examine the inter-relationships between diverse actors involved in all stages of vaccine delivery, identifies and enacts improvements to the regulatory environment, and promotes systemic transformation of those gender norms that prevent women from effectively benefiting from vaccine access and adoption. A gendered LVVC analysis identifies all stakeholders, systems and processes that would impact men and women smallholders’ individual and collective opportunities. Livestock value chain interventions have been used to design productivity improvements [19], but only most recently has a gendered analysis been employed to increase vaccine accessibility and adoption by women, with its follow-on family health benefits and empowerment [33]. This study aims at mapping and characterizing the CCPP-VVC in Machakos County to identify the key chain actors, to analyze the barriers women smallholder farmers face along the LVVC, and opportunities for their engagement. Qualitative data generated examined the gaps, and barriers that prevent women smallholder farmers from effectively accessing and adopting CCPP vaccination for their animals, as well as potential entry points for their participation. Using outcome mapping, a stakeholder analysis of the critical partners in the CCPP-VVC was done involving the vaccine manufacturers, vaccine importers, distributors, agrovets, public veterinary services, private veterinarians, local leaders, and farmers.

## 2. Materials and Methods

### 2.1. Description of the Study Area

**Figure 1:**
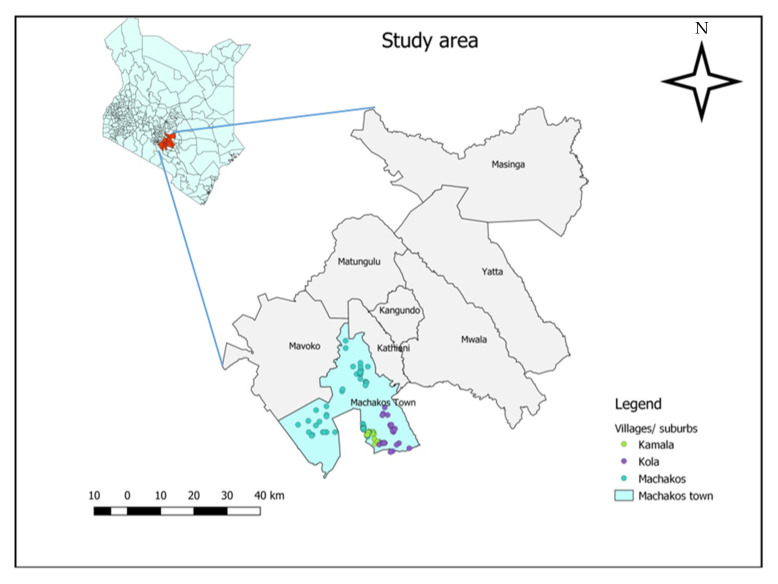
Map showing study area in blue.

The study was conducted in Machakos Town sub-county, Kenya, which is located 61.6 km southeast of Nairobi, Kenya’s capital city. Machakos Town sub-county has seven wards, from which Kola and Kalama wards were purposively selected because they own chickens and goats (Figure 1). The sub-county’s population is estimated to be 170,606. The climate is semi-arid, and the county has an altitude of 1000 to 2100 m above sea level. It lies between latitudes of 0.45′ S and 1.31′ S and longitudes 36.45′ E and 37.45′ E and covers an area of 6850 km^2^. The average rainfall ranges from 500–1300 mm, and the average temperature is 18–25 °C [34]. Subsistence agriculture is the main farm activity. Maize, in addition to such drought-resistant crops as sorghum and millet, is grown due to the area’s semi-arid nature. Most families own goats and/or chickens.

### 2.2. Data Collection Methodology

Our research was a cross-sectional study and used qualitative and participatory research methodologies. The strategy integrated gender analysis tools for action research in the LVVC. The USAID five domains of gender analysis was used as a gender analysis framework. This covered the following domains: (i) laws, policies, regulations, and institutional practices; (ii) access to and control over assets and resources; (iii) gender roles, responsibilities, and time use; (iv) cultural norms and beliefs, and (v) patterns of power and decision making. Thirty-nine key informant interviews (KIIs): 24 men and 15 women, were conducted with regulators at county and national levels, livestock extension workers, veterinarians, vaccinators, agrovet owners and attendants, vaccine manufacturers, distributors, suppliers, feed store owners and workers, local women farmers, community leaders, women group leaders, civic and public leaders, ecclesiastical elders, non-governmental organization leaders, and farmers along the LVVC. The KII were semi structured guides addressing farmer knowledge about chicken and goat diseases, gender and age disaggregated access to, control over, and benefits from resources; government policies and activities that affect vaccination of goats, and women’s roles and opportunities to increase benefits from LVVC.

Ten focus group discussions (FGDs) were conducted, with a total of 46 males and 76 female participants. Eight of the FGDs had separate male and female participants while two were mixed male and female participants. All FDGs were conducted using guidelines created in advance based on the USAID five domains of gender analysis [35]. A FGD guidebook was developed, and researchers pre-tested it prior to use. The FGDs focused on identifying gender roles, responsibilities, space, time use, and goat’s health problems and patterns of power and decision making, laws and policies as they impact women in the LVVC. The sustainable livelihoods assessment tool [36] was used with the eight sex-disaggregated FGDs to analyze the differential social, financial, physical, personal, and human control over assets for men and women in the community. The FGDs done with different stakeholders in the LVVC were useful to identify barriers to women’s access to delivery and distribution of vaccines. Data collection was enriched by holding focus group discussions with varying groups including women goat farmers, men goat farmers and mixed groups. Ranking exercises were done within the FGDs.

Outcome mapping (OM), a qualitative participatory process that allows different stakeholders to collaborate in a systems analysis was used to map and track critical changes in the cultural practices, organizational systems, institutional and governance policies, and the progress of stakeholders towards the goal of women’s empowerment in the LVVC. The OM tool helped to identify LVVC stakeholders and their formal and informal interactions. Three different stakeholder meetings were held, one with national level stakeholders that included vaccine regulatory bodies as well as vaccine manufacturers and distributors, and some deliverers, one with county level vaccine value chain actors including county administrative teams agrovet, public and private veterinarians, and one with community level stakeholders that included women group leaders and local community leaders and members. Through a facilitated process, stakeholders worked collaboratively to physically map out their roles and interconnections in the LVVC, support mechanisms, as well as existing systems including analyzing their current limitations and gaps, challenges and barriers that they face (both systemic and programmatic). The stakeholders identified challenges and opportunities for women’s participation, engagement, and ability to influence legal and governance structures within the LVVC. Focus meals, impromptu focus groups around a meal with randomly selected participants found in a semi-public setting (near a restaurant or market), were done. A free meal was provided as an incentive for people to share their stories and ideas. Group discussions took place over lunch and took 45–60 min. These groups were open to all community members of different genders, making space for those who otherwise may not have participated in the study. Jar voices were set up to capture people’s opinions in transit. They captured opinions and ideas of men and women about the gendered ownership of livestock, and participation in and constraints to vaccination of animals.

Jar voices were set up to capture people’s opinions in transit. They were done anonymously for both men and women to collect their views about the gendered ownership of livestock and participation in and constraints to vaccination of animals. Simple questions were written on flip charts and hung on walls of consenting drug shops for a day, then patrons were invited to write their answers and place them in a jar. The answers were collected and replaced with a fresh set of questions the following day. Jar voices helped to capture a community’s identity and voice in real time and space. A jar voice is a very effective tool for capturing voices of people who rarely participate in community gatherings, whose voices are smothered or are rarely selected by their community leaders but can be found in these spaces; the outside or lonely voice. In many patriarchal communities, women belong in this category. The jar voice questions were written in English and translated into Swahili. However, illiterate people and those who did not speak English and Swahili were assisted by the Agrovet staff to respond to the questions.

Table 1 below summarizes the data collection methodology.

### 2.3. Data Analysis

Data analysis included daily reviews of all data to identify and triangulate key findings. Data collected through key informant interviews, focus group discussions, and focus meals were audio recorded and transcribed verbatim in the local language (Kiswahili and Kikamba) and then translated into English for coding and analysis.

Inductive coding (a process whereby codes were derived from the data) of FGD transcripts were compared and contrasted and a comprehensive code book of thematic codes was developed for further data summation and analysis [37]. The coding was based on the five domains of gender analysis, as well as different frameworks such as the gender empowerment framework, chain empowerment matrix, Harvard analytical frameworks, and the Caroline Moser gender roles framework. Content analysis was used to examine patterns and interpret meaning [38]. Extracts and quotations were used as examples.

## 3. Results

The results of the study were organized into three subsections. Section 3.1 presents the CCPP vaccine value chain, different actors, and vaccine supply distribution. Section 3.2 focuses on barriers to vaccine uptake by both men and women farmers at the end-user level and for women along the CCPP-VVC. Section 3.3 presents opportunities and potential entry points for women engagement in the different nodes of the CCPP-VVC.

### 3.1. Mapping and Characterization of the CCPP Vaccine Value Chain and Its Actors

**Figure 2:**
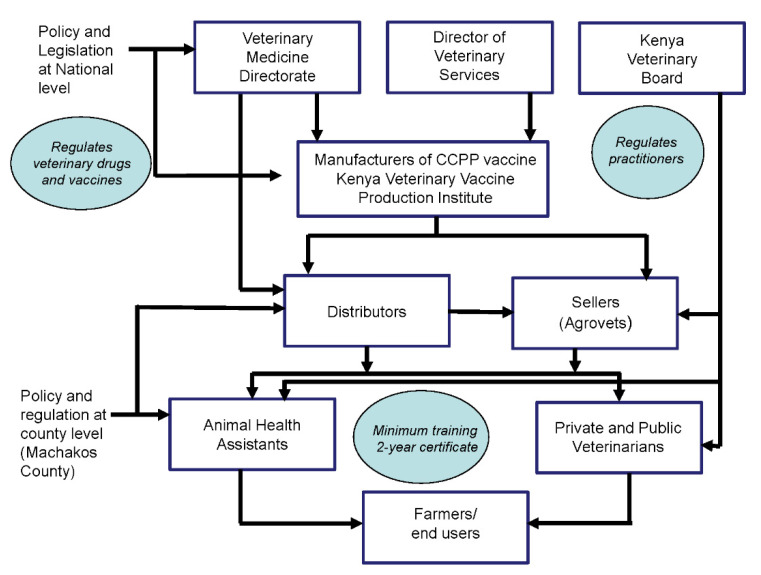
CCPP vaccine value chain showing legislation and distribution flow of vaccine from manufacturer to end user.

The CCPP vaccine in Kenya is regulated within a government distribution mechanism. The CCPP-VVC demonstrates a vertical linearity, with the value chain framework showing how a vaccine moves physically from the manufacturer to the end user, increasing in value with the nodes reflecting different actors along the chain. Along this linear chain, there are five types of actors: the policy makers and regulators; manufacturers of CCPP vaccine; vaccine distributors, who range from large companies to small private companies, and could include the local government and veterinary officers who operate private agrovet businesses; vaccine deliverers including public and private veterinary officers and animal health service providers at the county and sub-county level and vaccine users including commercial farmers, and smallholder goat farmers. Figure 2 shows the different LVVC actors and the regulatory and distribution flow of vaccine from manufacturer to end-user.

#### 3.1.1. The Legislative and Policy Framework for Vaccine Distribution

In Kenya, the state is responsible for the legislative frameworks that guide the manufacturing registration, distribution, and handling of the CCPP vaccine. An efficient regulatory authority is essential if quality standards are to be achieved and enforced. There are three government departments responsible for regulating vaccine development, distribution, and use: The Veterinary Medicine Directorate, the Directorate of Veterinary Services, and the Kenya Veterinary Board. The legislation part includes all policies that are related to or guide the use of vaccines, as well as the manufacture and distribution of vaccines both nationally and at county level. The Veterinary Medicine Directorate (VMD) regulates the manufacture, importation, exportation, registration, distribution, prescription and dispensing of veterinary medicines, drugs, and other animal health products in Kenya. The VMD operates at the national and county level. The VMD, therefore, oversees KEVEVAPI, which manufactures the CCPP vaccine. The Directorate of Veterinary Services (DVS) advises the national government on use of controlled vaccines and facilitates decisions on how and when to distribute vaccines. Counties make decisions on administration of these vaccines in their area of jurisdiction following advice provided by the DVS.

The Kenya Veterinary Board (KVB) ensures quality veterinary service delivery by veterinarians and animal health assistants in the whole country by regulating their registration, licensing, and field practice in Kenya through the Veterinary Surgeons and Veterinary Paraprofessionals Act, 2011 (Cap. 366) [39]. The board also inspects and accredits veterinary profession training institutions such as the University of Nairobi Veterinary School and the Animal Health Training Institutes (AHITIs). All veterinarians, whether in public or private sector, and animal health assistants, must be licensed by the KVB in order to provide veterinary clinical services or be allowed to own or operate veterinary pharmaceutical stores (agrovets). It is required that all agrovets must hire a licensed veterinary practitioner to operate in the country.

#### 3.1.2. CCPP Vaccine Manufacturers

The Kenya Veterinary Vaccines Production Institute (KEVEVAPI) was established as a government parastatal institution under Cap 446 of the laws of Kenya on 5 May 1990 [40]. This followed the disbanding of a joint venture between the Government of the Republic of Kenya and the Wellcome Trust Foundation of the United Kingdom. The Institute was created by merging the Vaccine Production Laboratory (VPL) at Embakasi, the vaccine production section at the KARI-National Veterinary Research Centre (NVRC) at Muguga and the vaccine section of Veterinary Research Laboratory at Department of Veterinary Services. KEVEVAPI manufactures, markets, and distributes the CCPP vaccine, CaprivaxTM, plus other veterinary vaccines in Kenya, and leads research, either alone or in collaboration with other research institutions, into new innovations of veterinary vaccines production [40]. CaprivaxTM is an inactivated Contagious Caprine Pleuropneumonia vaccine prepared from Mycoplasma capricolum capripneumoniae (Mccp). The vaccine is stored at between +2 °C and +8 °C (refrigerator) with a shelf life of one year. Once the vaccine bottle has been opened it must be used immediately, and any remaining quantity discarded. The vaccine is administered to animals of over 3 months of age via subcutaneous injection at the rate of 1 mL per animal. Revaccination should be done every 6 months.

#### 3.1.3. CCPP Vaccine Distributors

Distributors purchase vaccines from KEVEVAPI and distribute these vaccines to agrovets or veterinary practitioners. They also provide cold storage facilities while the vaccines are at the warehouse, facilitate cold chain transportation from the cold room to intended destination, and train agrovets on the proper storage and application of the vaccines. Most of the CCPP distributors are located in Nairobi, the capital city of Kenya. In Machakos County, the two largest veterinary drug distributors do not stock CCPP vaccines. Private and public veterinarians are also allowed to act as distributors if they have the appropriate cold chain.

#### 3.1.4. CCPP Vaccine Sellers (Agrovets)

Agrovets sell animal health products as well as agricultural supplies such as veterinary drugs, fertilizers, animal feed, veterinary supplies, and other farm supplies. In Kenya, agrovets are allowed to stock the CCPP vaccine if they have the proper cold storage facilities and are manned by a licensed veterinarian. As distributors above, they are also responsible for maintaining the cold chain. They advise buyers who are mostly veterinarians and animal health assistants on how to best use the vaccine (i.e., maintaining the cold chain and instructions on the general use of the vaccine). Agrovets play a very important role because they bring the vaccine closer to the consumers. They are one of the most important LVVC actors as they are licensed to stock any livestock vaccine. Many agrovets are owned or run by veterinarians or licensed under a veterinarian according to the Kenya Veterinary Surgeons’ Act. Agrovets are an important link in ensuring quality vaccine handling and issuing of vaccine use directives.

#### 3.1.5. Public and Private Animal Health Service Providers

This group consists of private and public veterinarians and veterinary paraprofessionals (animal health assistants). Since CCPP vaccine has to be delivered via injection, and requires a strict cold chain, only qualified practitioners with a minimum of a two-year college diploma are allowed to purchase the vaccine and inject animals. Besides vaccination, they provide training and extension services to the farmers.

#### 3.1.6. End-Users/Farmers

End-users of the CCPP vaccines include large commercial goat ranches as well as smallholder farmers including women farmers. They rely on the animal health service providers for vaccination services because the CCPP vaccine, which is administered via an injection, can only be handled by a qualified government or private veterinary service provider. They also rely mostly on government veterinarians to provide training and extension services on improving productivity of their animals. In Machakos, the CCPP vaccine is acquired either from the county government through the animal health services or from private sector veterinary officers. The efficacy of the vaccine depends on its handling. Most times, this is the point where vaccine quality and efficacy have been compromised as a result of wrong vaccine reconstitution (type and amount of water) or ineffective cold chain. Outside the official vaccination campaign periods officiated by the government, farmers could vaccinate their animals every six months if they chose to on their own or when an outbreak is suspected. In Kenya, CCPP vaccine is administered by registered veterinarians and paraprofessionals upon request by the farmers, who also have to pay for the vaccine and the veterinarian’s services.

### 3.2. Barriers to CCPP Vaccine Access at the End-User Level

Respondents identified barriers to CCPP vaccine uptake that were specific to small ruminant farmers at the end-user level. Men and women smallholder farmers prioritized these barriers differently. Men prioritized ineffective (fake) vaccines, lack of finances for purchasing vaccines, unqualified practitioners (quacks), slow veterinary officer’s response, and high cost of vaccination and vet services, in that order, as the top five leading hindrances to vaccine access. However, women ranked limited knowledge of goat diseases, high cost of vaccination/vet services, lack of awareness of government programmes, unqualified practitioners, and few veterinary doctors as their top five barriers. Table 2 below presents barriers ranked in order of importance for men and women.

#### 3.2.1. Inadequate Knowledge on Vaccines and Disease Management

Many women smallholder farmers said they lacked knowledge on goat diseases and vaccine use, resulting in low vaccine adoption. Some farmers were not aware that their animals could be vaccinated against CCPP. “*Is there a remedy against Mavui (CCPP) for animals? If there is something we can use to protect goats from lung disease, then it will be a big benefit for this community. Currently the solution is to slaughter sick animals*”, one woman farmer said. The rarity of CCPP vaccination was reported as a possible reason for the limited knowledge possessed by farmers. Farmers were unaware that they could regularly vaccinate against CCPP because vaccination campaigns were limited to when there was an outbreak. Farmers consulted veterinarians when animals were already symptomatic, which was too late for them to do anything. A public service vet said, “*When they come for treatment, it’s always too late because CCPP is not treatable. You can only prevent it through vaccination. The best you can do is symptomatic treatment. If reported early, prevention measures can be taken and those infected can be isolated as soon as possible to save the rest of the herd*”. He contended that since farmers sought services after manifestation of clinical signs, or sometimes after attempting to treat the animals with local remedies, information available at that time had little benefit “*I think the biggest issue is knowledge. Once people are empowered with knowledge, and they know that they can keep their animals safe by vaccinating, they will seek the vaccine*”.

Some private veterinarians, however, were not interested in providing information and teaching farmers to vaccinate on their own. “*I have to protect my business. If I provide the information to the farmer, he will be able to treat the animals on his own, and he will not need the services of a vet. I have to keep some knowledge on drugs and vaccines to myself, otherwise I will lose my livelihood as a doctor*”.

Participants agreed that more knowledge and training opportunities would create awareness about CCPP. A young woman in Kalama stated that: “*I don’t know the names of diseases, so cannot say anything. We need to be trained on them*”. The government department sometimes offered training, but this was infrequent, and was not accessible to everyone.

#### 3.2.2. Cost of Vaccine and Vet Services

The cost of veterinary services in addition to the cost of vaccines was seen as prohibitive for many women farmers. Most farmers reported that each service cost a minimum of five dollars initially to cover the transportation cost before including the cost of the treatment. They also claimed that many private vets charged per animal instead of charging a herd fee, making it unaffordable. The vets acknowledged that transportation was very expensive but argued that such costs were necessitated by the fact that they needed to hire transportation for farm visits. “*We do not have any service vehicles at the county level. When a farmer comes for help, she must provide transportation. Otherwise, I must hire a motorbike, and they need to pay for it*”. The number of animal health service providers were few and they had to travel long distances to provide services, making the cost more prohibitive for women smallholder farmers in the rural areas. This increased the cost-of-service delivery, and also discouraged many of the practitioners from going further into the rural communities to provide services.

Vaccine access is also hampered by packaging. The CCPP vaccine is usually packaged by KEVEVAPI in 100-dose vials costing 15 dollars (1500 Kenyan Shillings) each, which to many rural smallholder farmers, is very expensive. Most farmers own between 1 and 20 goats. Purchasing vaccines for 100 animals is, therefore, wasteful. This, plus the fact that the vaccine is expensive, makes it difficult for most farmers to afford the vaccine. One service provider said, “*small farmers don’t keep animals for commercial purposes and hence packaging of vaccines prevents them from buying the vaccine*”.

Desperate farmers are forced to purchase the 100-dose vials even though they do not need them and end up wasting their money and discarding the rest of the vaccine. Most agrovets consequently avoid stocking the vaccine because they do not sell very quickly. The high cost of the vaccine means fewer sales. This, in turn, constrains many private sector vets, as they cannot access the vaccine from any of the agrovets around Machakos. In one meeting, a private service provider said: “*I’m the one who goes everywhere in Machakos to treat animals. I know how much farmers suffer with CCPP. We do not have a single agrovet that stocks CCPP vaccine in this area*”. Private vets also reported that a lack of cold chain and the minimum dose quantity were limiting factors for them to stock the vaccine. Access was further limited by additional travel costs, if they had to obtain vaccines directly from KEVEVAPI, which is located about 100 km away.

During the discussions with women farmers, lack of finances was also raised as a limitation. Respondents reported that women farmers generally have no control over finances. This makes women dependent on their husbands as they cannot afford on their own to pay for vet services. A respondent from a pharmaceutical company concurred that most of their customers are smallholder female farmers but, unfortunately, some of them cannot afford to buy drugs nor vaccines. “*Even though most of the customers are women smallholder farmers, the majority cannot afford to buy vaccines because of financial limitations. Men control the money and women do not, they rely on men to provide*”.

The long distances from farm to vaccine purchase points were reported as one of the challenges especially for women, as it increased the cost of drugs and vaccines. Drug sellers, vaccine distributors and agrovets are mostly located in business centers too far away from the villages. A bus or motorbike from the interior of the village to Machakos costs $ 3–6. A woman from Kyakaili said that “*It takes up a lot of money. Three dollars to reach Machakos by matatu, otherwise you have to go by foot for three hours then you take a small bus to reduce the cost. We cannot afford to go to the town*”.

#### 3.2.3. Lack of Strategic Vaccination Plans and Information Programmes

Access to CCPP vaccines is constrained by its unique supply chain. The government only intervenes after an outbreak, based on demand from farmers. Consequently, the vaccines are often not only late but insufficient in quantity to meet the demand. According to the county director, the shortage of vaccines creates a real risk for farmers. It is recommended that vaccination against CCPP be repeated every six months for goats over 3 months old, and young ones at 2 months old. However, due to inadequate vaccine supplies, the government vaccinates once a year, or if there is an outbreak as reported by the farmers. In addition, government action is extremely slow, and vaccines arrive late usually after farmers have lost their animals. The vaccination process itself, once put into action, is prolonged, as a result of the lack of vaccines. “*Many times, they run out of vaccines, and we have to wait for a long time to receive the next supply*”, one farmer said.

According to the director of vet services, the Machakos County government provided around 8820 doses of CCPP vaccine in 2019. Only 7540 goats were vaccinated during that year’s vaccination campaign, a coverage of only 1.2% of the goat population of about 629,000. The campaign focused on the high-risk areas that experienced outbreaks. The government officers acknowledged that they were unable to help farmers because of inadequate supply of vaccines, and many farmers lost their animals as a result of that. “*CCPP can decimate the goat flocks anytime in Machakos. Therefore, the shortage and inconsistent availability of vaccine from the government is a real problem*”.

Respondents also complained of inadequate information regarding any government driven vaccination campaigns, despite the fact that county vets claimed to carry out publicity campaigns in local languages, going through local leaders, chiefs and assistant chiefs, churches, and schools. Most vaccination programs were published in the media, with very little access for local women in the community. Community members felt that vaccination teams were not adequately resourced to undertake proper publicity.

#### 3.2.4. Lack of Animal Health Service Providers

Considering that only qualified personnel are allowed to handle the CCPP vaccine, respondents reported that the insufficient number of veterinary doctors, the slow response when they were called, and infiltration by unqualified practitioners were major barriers. It is extremely difficult for KVB to track all practicing animal health service providers across the country to ensure they are licensed. They rely on the good will and integrity of practitioners, and on farmers to report if they suspect that people may be operating without a license. In some cases, especially in rural and remote areas, unlicensed people set up practices in the hope they will not be found out.

It was also noted that the county had inadequate numbers of animal health service providers and lacked resources to train or recruit more veterinary officers. The county has only four veterinary surgeons (one female) and eleven paraprofessionals (ten females, one male) to plan and execute vaccination campaigns, who are over extended. The Government is not able to replace those that retire or resign, forcing the director to rely on private animal health providers to vaccinate in cases of outbreaks. “*The staff are very few, for instance in Kalama we only have one staff who is trained but he is also the meat inspector. By the time he gets finished with inspecting meat he is too tired to take on any additional responsibility*”.

As a result, women have no option but to apply traditional methods of disease management for their animals in the absence of adequate vet services. Table 3 below presents statistics of staffing of the veterinary services in the whole of Machakos County. A single veterinarian serves over 28,000 households and supervises several veterinary paraprofessionals, each responsible for close to 4000 households.

#### 3.2.5. Cold Chain Maintenance and the Context of Fake Vaccines

Among the main challenges reported by both men and women was the maintenance of the vaccine cold chain by vaccine providers. This was also identified as a big problem for both private and government sector service providers. The board of animal health reported that to provide adequate clinical services availability of a cold chain was required to ensure vaccine efficacy. However, there were many cases of service providers using vaccines that had not been stored properly and were therefore not viable and effective. Animals still got sick after vaccination which upset farmers. Farmers referred to these as fake vaccines and refused service from providers as a result. Some private sector drug suppliers also reported that transport costs and the need for a cold chain due to the distance from KEVEVAPI to Machakos was a barrier to their business. KEVEVAPI recommends that CCPP vaccines be stored between 2–8 °C in a refrigerator, but most vets and agrovets do not have such storage facilities. Since individual households own very few goats, and cannot afford to purchase the vials as packaged, the CCPP vaccines are rarely stocked by agrovet shops, private vets, the government offices, or even non-governmental organizations that offer veterinary services. This is because they lack adequate cold chain facilities and qualified personnel to handle the vaccine. In Machakos County, the two largest veterinary drug distributors do not stock CCPP vaccines.

### 3.3. Barriers to Women’s Participation in Other Nodes of the CCPP-VVC

**Figure 3:**
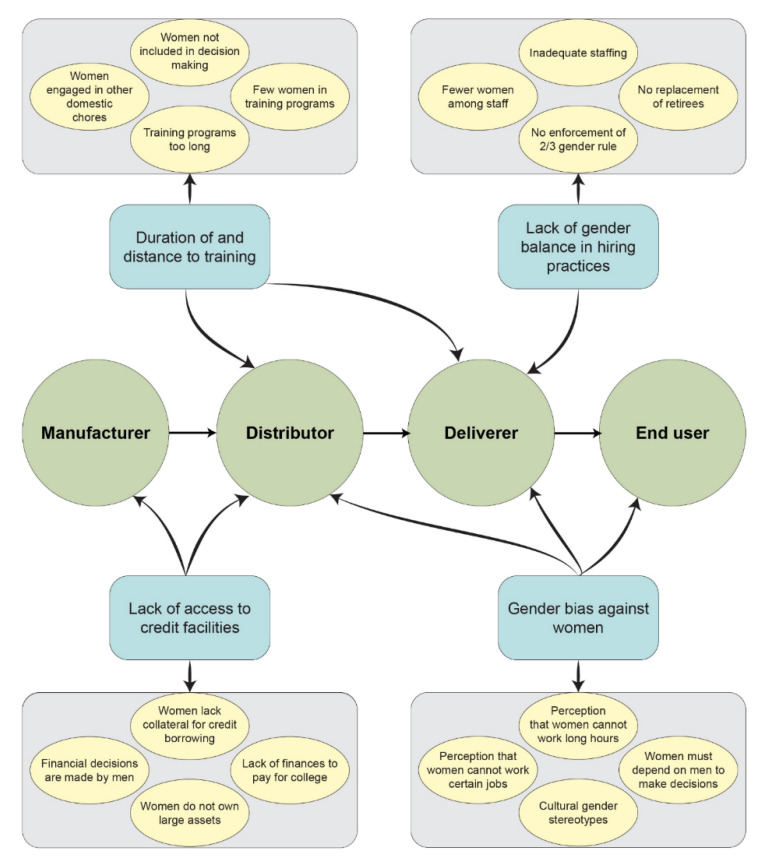
Barriers specific to women at different modes of the LVVC.

Along the different nodes of CCPP nodes, other barriers were identified as limiting women from fully engaging and contributing effectively to the LVVC. These included a lack of gender balance in the hiring process, few women in animal health training programs, lack of access to collateral for women interested in owning agrovet businesses, and gender bias against women. Figure 3 highlights these barriers and specific factors associated with them.

#### 3.3.1. Limited Number of Women among Staff Members in Veterinary Services

Government officials concurred that there was a gender imbalance among the animal health service providers in the county with few women. However, they stated that their priorities were not focused on the gender imbalance but on getting the work done, and so they hire whoever comes along, provided they meet the qualifications; most often, more men than women qualify. “*We recognize there are few women, but that is the nature of things. Unfortunately, we have very few ladies in animal health services*”.

A vet officer reported that even though there was a gender policy, it was not enforced. “*That is why they do not also care to engage women in the public animal health services*”. The county animal health director reported that not only are there few female employees, but also that a large number of staff are getting older and retiring without subsequent replacement.

#### 3.3.2. Limited Number of Women in Animal Health Training Programs

Animal health service providers and regulators agreed that the number of institutions training in veterinary or animal health sciences were few in the country, with even fewer females training in these institutions either as veterinary doctors or animal health assistants. In Kenya, only the University of Nairobi has been training veterinary doctors. Egerton University has only recently started producing veterinary doctors. Enrolment of students into science-based courses, and veterinary school in particular, is generally low with female enrolment accounting for one quarter of the total number since early 1980s. Although this ratio has improved to about two thirds currently [41], the completion rate is still low for females. It therefore follows that very few women were available to take up positions on offer in all sectors. Others concluded that it was because women were not interested in veterinary-related courses. The Director of Veterinary Services at county level reported that: “*shortage of lady candidates in job applications and internships demonstrated the fact that women are less interested in veterinary services, claiming that it is a male career. Their absence in the job market makes it difficult to consider gender balancing during recruitment*”.

One stakeholder maintained that gender awareness training should be provided to all men, including recruiters, so that they can be knowledgeable and understand that women, too, are capable of handling issues and making decisions on their own. One vet added that men do not allow their wives to go out for training, noting that as a big barrier to having female animal health service providers in Machakos.

#### 3.3.3. Length of Animal Health Training Programs Is a Barrier to Women

Barriers related to length of training limit women from serving as animal health providers. The respondents felt that the training favored men over women. Duration of training and distance to training institutions, particularly training in Nairobi, made these inaccessible for women. The veterinary board requirement of a two-year training period to be a vaccinator or animal health assistant was a condition that most married women found unfavorable, as most of them would not be allowed to be away from home for that length of time. The veterinary services director at the county, however, explained that two years is the minimum training period to acquire requisite vaccination competence, and that there was no alternative. Other barriers, according to the women, included lack of freedom to make training decisions on their own, preoccupation with domestic chores, and therefore lack of time for women to attend training. Men, on the other hand, cited financial constraints to send their wives to college for training as their number one barrier.

The county director of vet services reported lack of women recruits in the most recent veterinary internships in 2019 as a case in point, where they ended up recruiting more male vet officers. The other barrier was to do with policy on vaccination. The director reported that CCPP vaccination can only be done by a qualified vet, and the law is very vigorously enforced to prevent quacks from practicing.

#### 3.3.4. Cultural Expectations and Gender Bias against Women

Some of the male respondents argued that women were unable to work late hours, and certain jobs, such as delivering vaccines, sometimes involved working very late into the night, thus limiting women’s participation. Many stakeholders stated that cultural expectations for women to stay at home and to depend on their menfolk to make decisions resulted in low self-confidence among women and poor knowledge of how to access the information or support they needed. In some cases, livestock service providers held negative attitudes towards female farmers, calling them ignorant or incapable of using modern technology. One man observed, “*in some communities, norms and culture impede women from providing veterinary services. Even other women expect only men to be animal health assistants (AHA) and when they see a woman AHA, they do not trust them*”.

The lack of professional women working as veterinarians, or deliverers of information or vaccines, meant there were no role models for young girls, and very few showed interest in pursuing animal health careers.

#### 3.3.5. Lack of Financial Capital to Own Agrovet Shops/Businesses

The lack of financial capital was cited as a limiting factor for women, particularly those aspiring to develop a vaccine-related business, either as distributors or retailers. A woman running a small agrovet argued that such a business required a lot of financial input to (i) employ a veterinarian, (ii) buy supplies, and (iii) set up facilities including a cold chain. Several stakeholders agreed that this was, indeed, a big issue. Gender specific obstacles, such as lack of access to land and land rights, and inherent gender bias in the economic system, were cited as major roadblocks to women’s access to credit to invest in agrovet shops and livestock vaccines, putting women at a disadvantage. In addition, entrenched gender roles prevent women from participating in income-generating activities without their husband’s permission. A female vet running a small agrovet shop in Muumandu concurred further with the fact that lack of capital to attend veterinary training was, indeed, an obstacle she faced, and because of that she was unable to stock CCPP vaccine in her shop.

### 3.4. Opportunities for Women Engagement in the CCPP-VVC

Respondents identified opportunities for women engagement along the VVC.

#### 3.4.1. Job Opportunities as Animal Health Service Providers

Job opportunities in the public sector have been debated as an entry point to integrate women into the VVC. When asked, some of the policy makers including KVB, DVS and VMD raised the point that opportunities for government jobs in VVC are advertised openly so that all may apply, and those qualified are interviewed. “*Both women and men can apply when they meet conditions and qualifications set. However, all positions are competitive and not based on gender*”. Still, with many sub-counties and wards understaffed, there are expectations that soon places will be offered for many potential extension officers. On gender opportunities, the veterinary department respondent reported that opportunities for women in the directorate were increasing given the government’s directive on the two-third gender rule, implying that many women would be hired in the directorate if they had the right qualifications. “*I don’t think there are specific barriers that prevent women from participating as actors in the vaccine value chain apart from technical competencies. They might be hindered by lack of business skills*”.

Many farmers reported that they preferred women animal health attendants as vaccinators. When asked about training women to provide veterinary care, most farmers said that training local women on vaccination was very important because it would not only help them to treat their own and neighbors’ animals but, also, they would be able to buy drugs and vaccines at cheaper prices, reducing the cost of treatment and disease prevention for their animals. Other women leaders in Kyangala added that it would also increase availability of vet services to rural communities. Male farmers in Kalama also supported the fact that training would increase farmers’ knowledge of animal health.

There was a consensus that women animal health service providers were more trustworthy and preferred to men because “*they don’t tell lies, can’t administer a bad vaccine or drugs, don’t ask for a lot of money for services rendered the way men do*” and are available most of the time in the village. Women in Kyangala said that they have another advantage in that, culturally, women can attend/or access any household better than men. This helps them to spread messages very fast because traditionally people trust women. They are seen as being kinder and attend to several problems at the same time. All they lack are opportunities and space to practice. “*Women are very kind and understanding compared to men, our problem is lack of opportunities, and a space to prove that we are capable*”, said one woman. Everything considered, therefore, it is possible that training women as village vaccinators and service providers will improve both vaccine adoption and demand by their fellow female farmers, even at current service charges compared to their male counterparts.

Women mentioned that they were beginning to see differences and opportunities being offered to women to give their opinions in meetings. Women farmers in Kyangala admitted that: “*nowadays, things are changing. It is not like in the past when only men could talk. Even in different meetings they always give us time to express ourselves, and they are listening to us*”.

#### 3.4.2. Women as Livestock Managers and Decision Makers along the CCPP-VVC

Concerning the access for women to participate in VVC, several stakeholders including veterinary county officials, concurred that all along the VVC women are marginalized and yet women are the ones who often make the decisions to send their animals to be vaccinated and are thus the consumers of vaccines. Farmers and some stakeholders complained that the government does not make any effort to increase women’s participation in the VVC. “*Women are very good communicators, talking nicely and clearly, and any interaction about vaccination is well taken once done by a lady*”.

Government officials shared similar sentiments about women, acknowledging that whenever the vaccination team worked with women in the field their success was greater because most of the farmers are women, and they have greater capacity to convince others to vaccinate their goats. They stated that opportunities for women were getting better since enrolment into the veterinary medicine course at the university for the past few years has been approximately 50% male and 50% female students.

#### 3.4.3. Benefits of Training Women to Provide Veterinary Services

Respondents agreed that it would be a big step to train women farmers as animal health providers to create awareness about CCPP and help in its prevention in the area, since, as already mentioned, members of the community prefer women service providers. Women in Kyangala were encouraged whenever they saw a female animal health service provider working in senior government positions. A director of a private import company concurred that manual work was always given to men, while technical services including customer care and drug sales were given to females due to their soft skills. One female private sector agrovet owner admitted that they prefer women employees because they play more roles than men; they can clean the shop as well as sell drugs. Others reported that for them, recruitment is based on the choice of the agrovet owners. Private agrovet shops tend to have more women in the selling and customer care services and men in the manual/physical work and transport sector.

#### 3.4.4. Opportunities for Training in Animal Health and Husbandry

Women respondents claimed that opportunities for training are opportunities for empowerment. If they acquired knowledge in animal health and livestock husbandry they would be in a better position with increased livestock productivity, and increased herd sizes. They claimed that the more animals they owned, the more respected they were in the community, even by the men. Participants reported that training impacts women beyond their personal achievement by uplifting their living standards and that of their entire household. It also encourages the men to be more cooperative and give permission to their wives more when the training is in entrepreneurship, as the gains can be seen and enjoyed by all household members. Farmers agreed that training in livestock husbandry, disease management and entrepreneurship would improve the livestock sector and encourage women to keep animals healthy for economic gain.

## 4. Discussion

In this study men and women actors and informants along the CCPP-VVC prioritized and ranked barriers to vaccine access and adoption very differently. Men prioritized ineffective (fake) vaccines, lack of finances for purchasing vaccines, unqualified practitioners (quacks), slow veterinary officer’s response, and high cost of vaccination and vet services, in that order, as the top five leading hindrances to vaccine access. However, women ranked limited knowledge of goat diseases, high cost of vet services, lack of awareness of government programmes, unqualified practitioners, and few veterinary doctors, as their top five barriers. There is need to acknowledge that smallholder livestock farmers are not a single homogeneous group. Men and women smallholder farmers face different concerns, prioritize barriers differently and, therefore, approaches to developing interventions must take that into account. These differences contribute to inequalities and power imbalances and allow us to visualize the highly gendered nature of the vaccine value chain, which explains who in the value chain can access or control information. Women feel that they do not receive information on livestock diseases and vaccination, whereas men do not find this a problem and are concerned by “fake vaccines”, a situation that tends to indicate that they know about vaccination and have already some concerns about its efficacy. This is because vaccination information was relayed through veterinary personnel to the public administration chiefs, assistant-chiefs, and village elders, public announcements, and the radio; avenues that are more accessible to men than women. This information aligns with Serra et al., which discusses the significantly gendered and intersectional livestock vaccine value chains in Uganda, Senegal and Nepal, and the impact on PPR vaccine access [33]. Using gender transformative approaches that address both fundamental causes and consequences of gender inequality, an approach that looks at both the social context that contributes to existing the inequities and to their persistence as well as the enhancement of the opportunities in terms of information access, resources, technologies and environment, can lead to better development outcomes for women smallholder farmers.

Men and women engage in livestock farming/keeping for different purposes and, therefore, targeted interventions need to be developed with this in mind. A gendered perspective and analysis on barriers to livestock production and disease prevention (i.e., mitigation, adaptation, policy development) decision-making needs to be applied. Understanding the different barriers women smallholder livestock farmers face as opposed to men is critical, especially as in this case and elsewhere, women are already relegated to the end user node of livestock value chain [33].

Gender differences should push policy makers in developing gender-transformative and more informed programmes to enhance livestock farmers in general, and women’s welfare and participation in particular. Using gender transformative approaches that address both fundamental causes and consequences of gender inequality, an approach that looks at the social context that allows the inequities to exist, as well as the enhancement of the opportunities in terms of information access, resources, technologies and environment, can lead to better development outcomes for women smallholder farmers. Considering gender differences in livestock management and production and reflecting them into livestock programs and policies is of utmost importance, especially considering that such differences are often underpinned by social and cultural norms and stereotypes [33,42].

CCPP vaccine costs are influenced by the cost of the vaccine itself, long distances traveled by both farmer and veterinary service providers, compounded by lack of transport infrastructure, and lack of a cold chain to bring the vaccine closer to the people. This, and the fact that women in poor rural Machakos lack mobility (as they cannot ride or ride on motorbikes; the only means of transport available in the difficult terrain of the study area), lack autonomy and decision-making power regarding goat keeping, poses an enormous problem for goat farmers, particularly women, in Machakos. This is exacerbated by the fact that the government is the sole supplier of the CCPP vaccine. Eradication of contagious and endemic diseases becomes a challenge when the government is the sole supplier of medication and vaccine, as is the case with the CCPP vaccine in this study, and especially so if such government strategies do not take into account the existing disparities among the livestock holders [33], forcing the needs of the vulnerable individuals to always remain unmet. Studies done on similar diseases insist that eradication requires involvement of all stakeholders in the LVVC, including the private sector, NGOs, public sectors, and farmers themselves, in a well-structured stakeholder engagement [43,44], which was the approach of this study. However, LVVC partners require capacity and resources to understand how to apply existing national commitments to gender equality policies to their internal management, and then to implement, enforce and monitor them.

The case is worsened when the production, and or supply, of the vaccine is not adequate, as in the case of CCPP in Machakos. Farmers complained all the time that even when the government organized a vaccination campaign, the vaccine was not enough to cover all the animals, and many of them had to go back home without vaccinating their herds. The government’s strategy also fails because not only is the free vaccine coverage inadequate to cover the demand of the county, but the vaccine also reaches the farmers as a treatment measure rather than a preventive measure, since the service providers often arrive after an outbreak has occurred. Consequently, more farmers lose their flock before and after vaccination. This experience has a negative impact on the farmers such that the next vaccination campaign will have a poor response resulting in a low number of vaccinations. This low level of involvement of farmers in vaccinations results in minimal vaccine adoption and, consequently, failure of eradication efforts. Due to its high cost, very few farmers purchase the vaccine. The end result is a low quantity of vaccine with the stockists. This was demonstrated very well in this study where agrovet shops declined to stock CCPP vaccine because of its low demand [44]. This phenomenon has also been seen in other countries in Africa [25] and resulted in reduced vaccine production and stocking as suppliers are not willing to stock products that are in low demand, resulting in income loss, especially for products with limited shelf life [11].

Additionally, compared to other livestock vaccines, the CCPP vaccine is relatively expensive, costing 15 dollars for a 100-dose vial. Combined with short lived immunity and need for increased vaccination frequency, the CCPP vaccine has limitations in a mass vaccination program [45]. Another study done in Kenya found that costs emerged as the greatest barrier to vaccine adoption. Cost is a critical determinant of vaccine uptake, since in households where livestock vaccination costs are higher than available disposable income, farmers may forfeit vaccination or only have some of their animals vaccinated [46].

A combination vaccine of CCPP with another agent, such as Rift Valley fever, Peste des petits ruminants virus (PPR), foot and mouth disease (FMDO) sheep poxvirus and enterotoxaemia, could reduce costs for individual farmers as well as encourage mass scale immunization campaigns [45,47]. Furthermore, traditional vaccines are not without fault. Their efficacy for instance can be suboptimal with certain pathogens, and safety concerns have been raised with live attenuated vaccines [48]. This underlies the focus within the last three decades on using synthetic genomic techniques based on genetically modified organisms to identify novel vaccine candidates in veterinary medicine [48]. Such vaccines are becoming acceptable around the world, and in East Africa they will circumvent several of the flaws associated with classical vaccines. The East African community is identifying opportunities to harmonize and synergize policies across the region to enable usage of products across multiple countries once they are approved in one country. This means that a vaccine registered in one country would also be registered in other countries, decreasing costs and delivery bottlenecks.

A recent study on the adoption of goats’ vaccine in Kenya demonstrated that CCPP is ranked by animal keepers as one of the highest priority diseases in livestock, higher than Contagious Bovine Pleuropneumonia, and yet the vaccine is still inaccessible to farmers even during an outbreak, and quite expensive when compared to other animal vaccines [44]. Many smallholder women farmers believe that the government places greater emphasis on cattle, rather than small ruminant health care, and that small ruminants are typically seen as having a secondary status, along with their perceived primary keepers, who are mostly women. According to the director of vet services, the Machakos County government provided around 8820 doses of CCPP vaccine in 2019. Only 7540 goats were vaccinated during that year’s vaccination campaign, a coverage of only 1.2% of the goat population of about 629,000. Renault Veronique’s research in the ASAL zones of Kenya demonstrated that in a normal herd of 100 goats, the annual economic losses due to CCPP was projected at around 1712.66 € per year [11].

The Kenya government and its supporting institutions are responsible for the development of strategies and legislative framework that guide the manufacture and registration of vaccines, including access of these vaccines to women small-scale farmers. The state is therefore accountable for the implementation of the relevant control measures. CCPP vaccine distribution is controlled and regulated by the government. The tensions lie in diseases that are important at the farm or village level but not prioritized for state intervention. For instance, CCPP is notifiable, meaning it must be reported to the state, but all regular control measures including vaccination are the responsibility of the owner/s, unless there is an outbreak. Government regulations play a key role in vaccine delivery to all end-users, but current public funding and policies support cattle production, with minimal recognition of the importance of goats in sustaining family livelihoods. Vaccine uptake is a complex process that requires buy-in from men and women farmers, veterinary departments, county/district and national governments, and vaccine producers. It ultimately depends on the social context and must respond appropriately to the power dynamics in the household, community and across the entire livestock vaccine value chain. We have to recognize intra-household dynamics, control over resources and who decides what. Gender roles and relations in the households intersect with positions, relationships and responsibilities, which must be understood to create truly transformative projects that raise the position of women relative to men. The gender-blind history of livestock development projects has all too often resulted in increasing the workload of women without empowering them. Coupling interventions that enhance the equity of the social environment in the LVVC and technical components such as training, and provision of the cold chain can enhance women’s instrumental agency and lead to better outcomes for women, men, their families and communities.

Interviews with veterinary services providers and other stakeholders revealed that the involvement of women in vaccination campaigns has a positive impact on the community. Farmers reported that the presence of a woman vet officer in the livestock service stimulated more farmers to seek vet services, and that female vets were preferred over their male counterparts. They recognized that women’s involvement in the livestock sector results in better results, translating to better vaccine uptake. Respondents stated that whenever the CCPP vaccination teams worked with women in the field their success was greater because most small-scale goat farmers are women and have greater capacity to convince others to vaccinate their goats. A study done in Mali on the vaccination of PPR supports the findings of the present study, as it demonstrated that the incomplete involvement of women in vaccinations was considered as one of the main challenges in the implementation of vaccination [25]. The present study further corroborates other findings that women are caretakers of their animal’s health in different societies [49,50], suggesting that they should not be sidelined in activities geared towards the promotion of vaccine uptake. Creating more entry spaces for women along the LVVC, and opportunities that foster a vertical shift of women along the LVVC from end-users of the vaccines and veterinary drug shop attendants to becoming women entrepreneurs, animal health service providers and decision makers at the various nodes of the chain, is critical for improved livestock productivity in Machakos county.

There are many opportunities for women to engage in the CCPP-VVC as end-users, entrepreneurs, deliverers, and even at the vaccine distribution level. However, this needs a deliberate effort on the part of the policy makers and regulators to enforce gender-responsive policies and to deliberately examine reasons why, even with those policies such as the government’s two-third gender rule concept, which states in article 81(b) of the Kenyan Constitution, that “Not more than two thirds of the members of elective or appointive bodies shall be of the same gender”, women are still not being hired as animal health service providers. Most of the relevant livestock policies are written in gender-neutral language, but their effects are frequently different for men and women. Currently, the policies on vaccine development and distribution in Kenya seem to be gender neutral. Moreover, there is no point along the value chain where there is a consideration for gender dynamics, i.e., manufacturing a vaccine that women can use easily, considering that women tend to own fewer animals and thereby packaging the vaccines in smaller vials to target smallholder farmers, focusing on producing vaccines that do not require a cold chain, and making a deliberate effort to recruit women as animal health service providers.

Other opportunities include reviewing the training program for animal health assistants to make it more time and user friendly for women. The current program is based on systems established in the 1980s when people were required to go away for two years to attend college. Modifications can be done to create interrupted programs that allow for short periods of didactics with the rest of the time spent in the field through an experiential learning program. Programs like these are successfully being implemented and reflect the best learning practices for most field practitioners. If away from home training sessions could be reduced to one month, three times a year, with a shift to internships and practical experiences, more women could participate. The empowerment of women along the VVC needs to be continued through increased access to education, information, training in animal healthcare, and ownership over assets and land.

## 5. Conclusions

This study exposes gender-related issues in the livestock vaccine value chain. It highlights the constraints and gaps in the current CCPP-VVC against which gender based cultural and non-cultural barriers exist to constrain and limit women farmers from accessing services related to livestock keeping and vaccines. This study demonstrates the existence of barriers encountered in laws, regulations, culture, practices, access to finances and other services for small scale farmers seeking to rear goats and to access the CCPP vaccine as end-users or play other entrepreneurial roles along the vaccine value chain.

Women smallholder farmers are still facing many challenges to access CCPP vaccines including high cost of vet services, lack of finances, knowledge and awareness on disease management, vaccine availability, and poor vaccination programs. On the part of the government, lack of resources, inadequate response and planning for vaccination campaigns, poor enforcement, or absence of gender-responsive policies, are the main factors preventing women smallholder farmers from accessing vaccines. However, opportunities exist that can be used as women’s entry points in the LVVC. Our results show that in spite of the lack of gender balance in veterinary services, both male and female farmers have preference for women veterinarians, reportedly because they are considered more skillful, honest, and reliable enough to deliver correct and viable vaccines and good quality drugs. They also are more accessible, and willing to engage.

The significance of women in smallholder livestock farming needs to be firmly established as a targeted policy imperative and as a part of a wider food-security strategy. Understanding the behavior, interests, inter-relations, and intentions of different LVVC stakeholders can be used to assess the influence, resources and effect these stakeholders have on women’s entry and participation as key players in the LVVC. Vaccine uptake is a complex responsibility shared by men and women farmers, veterinary departments, county/district and national governments, and vaccine producers. Women’s participation and influence in the LVVC translates into their ability to exercise decision-making and other powers in wider domains such as the creation of gender-responsive laws and regulations related to disease control, increased access to education, information, training in animal healthcare, and ownership over assets. Women also need to be empowered to take on leadership positions within rural livestock-farming communities and to play a role in intra-household and communal decision-making. They need to be included in regulatory policy making and enforcement.

Women smallholder goat farmers need to be well linked to the LVVC for them to maximize the value chain benefits. Whenever the public sector works with more women, their opportunities increase, and women’s roles in the society are recognized and appreciated. We conclude that if more resources, information, and training is available to women smallholder farmers, including opportunities as veterinary service providers, there would be increased adoption of CCPP vaccine and women’s visibility in the VVC as actors would increase translating into improved livestock productivity, better livelihoods, and agency. A holistic and sustainable model that focuses on systemic transformational change within the animal health sector to value women’s contributions and support their empowerment, is essential. Effective gender transformative approaches require political commitment to changing the status quo, allocation of resources, and adequate time for reflection and change.

## Figures and Tables

**Table 1:** Tools used for data collection.

Tools Used	No. of Events	No. of Participants		
		Male	Female	Total No. of People
Key informant interviews (KII)	39	24	15	39
Stakeholders’ meetings (SM)	3	22	14	36
Outcome mapping meeting (OM)	2	11	19	30
Focus group discussions (FGD)	10	4 (46)	6 (76)	123
Focus meals	6	12	14	26
Jar voices	10	-	-	122

**Table 2:** Identified barriers for men and women smallholder farmers to access CCPP vaccines listed in order of importance.

Barriers to CCPP Vaccine Access	
Men	Women
Ineffective (fake) vaccines	Limited knowledge on goat diseases
Lack of finances for purchasing vaccine	High cost/charges for vaccination/vet services
Unqualified practitioners (quacks)	Lack of strategic vaccination plan and awareness about government programmes
Slow response by vet officers	Unqualified practitioners (quacks)
High cost/charges for vaccination/vet services	Few veterinary officers
Long distance to vaccine access points	Lack of finances for purchasing vaccine
Few veterinary officers	Long distance to vaccine access points
Limited knowledge on goat diseases	Ineffective (fake) vaccines
Lack of strategic vaccination plan and awareness about government programmes	Wrong advice from vet officers
Wrong advice from vet officers	Slow response by vet officers

**Table 3:** Staffing of the veterinary services in Machakos County.

Service Providers Levels	Sex	Public Sector	Private Sector	Both Total
Veterinary Doctors	Men	3	9	12
	Women	1	1	2
	Total	4	10	14
Animal Health Assistants	Men	1	52	53
	Women	10	25	35
	Total	11	77	88

## Data Availability

All relevant data are within the manuscript and Table S1.

## Supplementary Materials

The following supporting information can be downloaded at: https://www.mdpi.com/article/10.3390/ani12081026/s1. Table S1: Supporting Information files.
