# Supplementary material for: Gendered Barriers and Opportunities for Women Smallholder Farmers in the Contagious Caprine Pleuropneumonia Vaccine Value Chain in Kenya
Source: Animals (Basel). 2022 Apr 14;12(8):1026. doi: 10.3390/ani12081026 (PMC9031503; doi:10.3390/ani12081026)
Supplement: Supplementary file 1 [file animals-12-01026-s001.zip › animals-1619662-supplementary.pdf]

Table S1. Supporting Information files.

Domains	Subthemes	Codes
1. Laws, Policies, Regulations and Institutional Practices (The Chain Empowerment matrix)		Gender / identity of government veterinary workers
	Institutional practices	Influence of institutional practices
		Cooperation
		Private sector
		Religious practices
		Support/aid programs
		Government engagement
	Policy interventions	Intervention (policy, programs, etc.)
	Veterinary policy	Veterinary role in vaccine administration/VVC
		Practices/policies to include female veterinarians in school
	Vaccine Policy	Limited vaccines registered by law
	Accountability of government	Local/public administration
	Regulations	Disposal of dead animals
	Barriers	
	Opportunities	Cooperation
2. Access to and Control over Assets and Resources (including income, employment, and assets such as land) (Harvard Analytical Frame-work, Gender Empowerment matrix)	Access	Access to resources
		Access to finances
		Access to opportunity
		Access to education
		Access to social assets
		Access to human capital
		Access to physical environment
		Access to personal assets
		Access to livestock
		Access to technology
		Access to leadership positions for women
	Barriers to access	Drivers license
		Transportation / moto not comfortable for women
		Livestock mortality as a barrier to economic success
	Control	Control over human capital (skills one has, knowledge and skills)

		Control over resources
		Control over social assets (networking, groups)
		Control over finances
		Control and decision making over family
		Control and decision making over goats
		Control over personal assets
		Control over physical environment
		Control over opportunities
	Barriers to control	
	Opportunities	
3. Gender Roles, Responsibilities and Time Use (Caroline Moser Gender Roles Frame-work)		Dependence of women on men
		Ability to multitask
		Women and men spend their time differently
		Gender roles
	Allies	Male allies
	Time availability	Time availability for women
	Animal type ownership	Chicken
		Goat
	Biology	Pregnancy
		Access of women to agrovet positions
		Difficulty to adopt roles that are not usual for this gender
	Barriers	Job opportunities
	Opportunities	Prioritizing girls vs. boys
		Gender-based opportunity
4. Cultural Norms and Beliefs (Force-field analysis)		Women are weak/have no control
		Hanging cultural norms
		Impact of misogyny on women
		Community norms and beliefs
		Consequences of norms and beliefs
		Judgement of women based on decisions to use resources
		Men's perceptions of women buying drugs
		Influence of support program on family culture and gender norms

	Relationships	Power struggle
		Marital conflict
		Comparing and measuring success of wives
		Relationship/communication between husband and wife
	Barriers	
	Opportunities	
		Gender norms and becoming female importer
		Female role models encourage more female involvement
		Prioritizing education for girls vs. boys
		Female Farmers cultural norms and use of vaccines and raise animals
		Auto marginalization
		Women as animal keepers
		Girls preference in term of education
5. Patterns of Power and Decision-making (Household decision-making models- Unitary and collective/cooperative conflict models)	Decision roles	Women's decision making and control over family
		Decision making power to choose to become female veterinarian
		Opinion about why men allow women to have control and decision-making power
		Strategies to influence husband's control and decision making
		Influence of communication on men's control and decision making
		Barrier to women having control and decision-making power
		Influence of culture and beliefs on men's control and decision making
		Strategies to influence husband's control and decision making
6. Education, training and skills	Training	Benefits of training
		Trained community members
		Benefits of training women to provide veterinary care
		Access to training for women
		Training on vaccination
		SMS and technology training
		Empowerment training
		Training of advisors
	Education	Access to education for women
		Benefits of education/training women
		Education for girls
	Knowledge	Lack of knowledge on vaccine and vaccination

		Lack of Knowledge on livestock health and management
	Barriers	Access to information barrier
		Barriers to women participating in training and education
	Opportunities	Opportunities for training to improve access and control over assets
	Sensitization	
	Group organization	Group structure and organization
		Culture of community groups based on gender structure
		Group formation
		Benefits of participation in groups
		Building group capital
		Challenges for women's groups
		Group activities
		Aspirations for participation in groups
		Goat ownership facilitated by groups
		Consequences to group membership
		Influence of group membership on family culture
		Control over group membership
		Negative consequence of group membership
7. Livestock ownership, disease and prevention	Vaccination	Goat vaccination
		Cold chain
		Access to goat vaccines
		Access to drugs / vaccine
		Vaccine packaging
		Type of vaccines
		Cost of vaccine
		Source of vaccines
		Lack of vaccines
		Access form women to VVC
	Veterinary services	Access to veterinary services
		Gender/identity of veterinarian
		Distance to livestock health services
		Lack of veterinary extension services
		Equipment (storage transport)

	Disease management	Herbal and other treatment modes
		Poultry disease management
		Goat disease management
		Causes and symptoms of poultry diseases
		Causes and symptoms of goat diseases
		Alternative NCD/ CCPP treatment
	Ownership of livestock	Goat ownership facilitated by groups
		Goat ownership
		Challenges to raising goats
		Barriers to raising goats: poverty
		Goat breeds
		Barriers to goat ownership for women
		Goat husbandry and management practices
		Calendar for chicken mortality
		Benefits to raising chickens
		Control over livestock
		Barriers to access and control over livestock
		Opportunities for training to improve access and control over assets
		Mortality of goats
		Mortality of chickens
8. Empowerment		Desire for empowerment
		Women's empowerment
		Priorities of empowered women
		Benefits to empowering women
		Barriers to empowerment for women - cultural beliefs
		Opportunities to improve respect of women
		Control over self
		Self esteem / women’s confidence
	Barriers	Lack of role models
	Opportunities	Training
